# Vertical and geographic distribution of copepod communities at late summer in the Amerasian Basin, Arctic Ocean

**DOI:** 10.1371/journal.pone.0219319

**Published:** 2019-07-11

**Authors:** Yan-Guo Wang, Li-Chun Tseng, Mao Lin, Jiang-Shiou Hwang

**Affiliations:** 1 Third Institute of Oceanography, Ministry of Natural Resources, Xiamen, Fujian, China; 2 Institute of Marine Biology, National Taiwan Ocean University, Keelung, Taiwan; 3 Center of Excellence for the Oceans, National Taiwan Ocean University, Keelung, Taiwan; 4 Collaborative Innovation Center of Deep Sea Biology, Hangzhou, China; 5 Department of Biomedical Science and Environmental Biology, Kaohsiung Medical University, Kaohsiung, Taiwan; University of Guam, GUAM

## Abstract

Zooplankton plays a pivotal role in linking primary production to higher level consumers in the food webs of marine ecosystems. The distribution of zooplankton is affected by general water conditions, monsoons, currents, and spatial and temporal factors. In the Arctic Ocean, the sea surface is naturally covered with ice. Under ice, water masses interplay to create complex environments that facilitate the transport and distribution of zooplankton, thus altering community structures at geospatial and vertical scales. The present study investigated the species composition and copepod community structures by using geospatial and multiple depth scales, and using multivariate analyses to evaluate the relation of sampling stations and layers. During July–August 2010, zooplankton samples were collected and the temperature and salinity of seawater measured from three stations in the Canada Basin and two stations in the Makarov Basin of the Arctic Ocean (maximum distance of approximately 1400 km). A total of 55 copepod species (including 25 species that were solely identified to the generic level) and 7 taxa of copepodites, altogether belonging to 28 genera, 11 families, and 2 orders were identified, and significant differences were detected in copepod community structures among sampling strata and at geospatial scales. Numerically, *Calanus hyperboreus*, *Calanus* copepodite, Calanoida copepodite, *Calanus glacialis*, and *Metridia longa* were the most dominant species and taxa. At the local scale, copepod compositions responded differently at each of the sampling stations. At the geospatial scale, the distance between stations MS03 and ICE explained variations in the pattern of dominant species and of copepod community richness. Our study demonstrated varied spatial distribution which indicates that (1) the abundance of copepods at 0–200 m was significantly higher than at other strata, (2) vertical strata affected the distribution of copepod communities, and (3) the interplay of North Pacific and Atlantic waters shaping the copepod assemblage structure at geospatial scales in the Arctic Ocean. The results of our research provide base data for Arctic zooplankton biodiversity and biogeographic distribution.

## Introduction

The Arctic Ocean is a unique environment with remarkable seasonality of light availability and its year-round ice cover. With its unique habitats, the Arctic Ocean is one of the most sensitive marine ecosystems susceptible to global changes [1]. Microwave remote sensing data have revealed an accelerated decrease in Arctic sea ice cover in recent years [2, 3]. The lowest Arctic sea ice coverage was recorded by satellite on 13 September 2012 [4]. The decreasing areal coverage of sea ice in recent decades has increased the absorption of solar radiation, resulting in a warming of the ocean surface [5–7]. Warming and ice loss affect the radiative balance of polar waters, requiring additional freshwater input that would presumably disrupt the global conveyor belt [8, 9], and change the phenology and species composition of autotrophic and zooplankton communities. Therefore, studies need to be conducted on the ecology of the three major realms of the Arctic Ocean: the sea ice, water column, and sea bottom.

Early studies of zooplankton in Arctic Ocean waters have been restricted to the sampling methods in coastal waters and have been conducted on drifting platforms [10]. Variable taxonomic results of zooplankton in the Barents, Kara, Laptev, Chukchi, and Beaufort seas were reviewed by Smith and Schnack-Schiel [11]. The taxonomic composition [12, 13] and life history of the larger-sized and common species of copepods have received most attention in studies on the faunistic composition of zooplankton because of the higher abundance and ease of capture of those copepods [14, 15]. In the Arctic Ocean, large endemic calanoids account for 50%–90% of the mesozooplankton biomass [16]. Copepods play a key role in the transfer of primary production to vertebrate predators at top levels of the Arctic marine food web [16–18]. Thus, studies have focused on the distribution pattern, community, and feeding behavior of copepods [19]. Arctic copepods reserve lipids exceeding 60% of their dry mass, caused by their efficient grazing of ice algae and phytoplankton during the spring bloom [17, 20]. The accumulation of lipids sustains copepods through the long winter without feeding and represents a crucial food source for other zooplankton and pelagic fish species [17].

A number of earlier reports have explored the distribution and species composition of copepods in the Canada Basin [21]. Previous studies have mainly collected samples from shelf areas and in margins of drifting ice in the deep basin areas [22]. Recently, summer cruises collecting samples from icebreakers have contributed to our understanding of the distribution of zooplankton in the northern part of the Canada Basin [23, 24]. Thus far, the distribution and species composition of zooplankton across the whole Canada Basin have not yet been explored [14, 25, 26]. Therefore, surveys on planktonic copepods conjunctive to the rapid changes in ice and marine ecosystems were conducted in the Canada Basin. The objectives of the present study were to: (1) analyze the vertical distribution of copepod community structure associated with water masses, (2) evaluate copepod diversity, composition structure, distribution patterns in their geospatial variability, and their depth distribution, and (3) investigate the effect of interplay water masses in the Canada Basin.

## Materials and methods

### Study area

**Fig 1:**
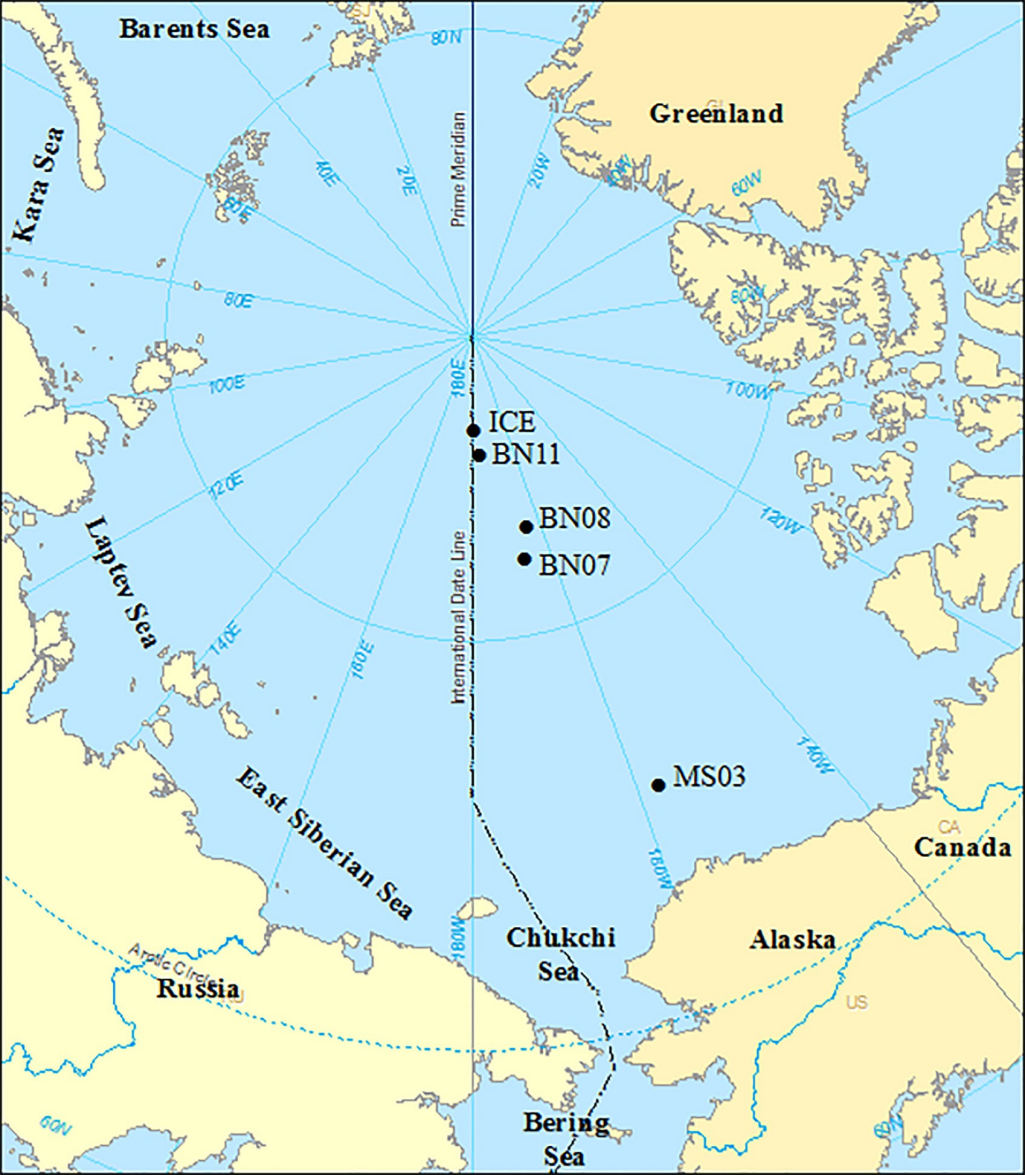
Map of the study area and location of sampling stations in the Amerasian Basin during the period from 28 July to 18 August 2010.

The Arctic Ocean, an area covering approximately 1.4 × 10^7^ km^2^, is surrounded by land (Fig 1). The Lomonossov Ridge (sill depth of 1400 m) acts as a dispersal barrier of deep-water currents [27, 28], dividing the Arctic Ocean into the Canada or Amerasian (maximum depth of 3800 m) and Eurasian (maximum depth of 4200 m) basins. The Amerasian Basin is composed of the Canada Basin and Makarov Basin, which are divided by the Alpha–Mandeleev Ridge. The Nansen–Gakkel Ridge divides the Eurasian Basin into the Nansen and Amundsen Basins. Five sampling stations were selected to investigate the community composition of copepods in the Amerasian Basin, between 74°4.2´–86°55.2´N and 157°18´–178°21.6´W (Fig 1). The geographical coordinates, date and time of sampling, and depth strata sampled are shown in Table 1. The stations included three stations (MS03, BN07, and BN08) in the Canada Basin and two stations (BN11 and ICE) in the Makarov Basin. Station MS03 was located at the edge of the Chukchi Plateau, in the southern Canada Basin. Stations BN07 and BN08 were located in the northern part of the Canada Basin, which is affected by North Pacific waters from the Bering Strait [29]. Stations BN11 and ICE were located at the Makarov Basin, which is influenced by Atlantic and summer Pacific water masses circulating at different depth levels [6, 30]. During the investigation period, most of the research area was covered by first-year ice with a mean thickness of approximately 94–114 cm. The ice was in a state of rapid melting and retreat. The recorded air temperature was between −0.5 and 0°C in the research area, similar to previous reports of Arctic Ocean cruises [31]. The water column structure in the Canada Basin was more complex than that in the Eurasian Basin because of the extreme temperatures found in the zone between the surface mixed layer and the main thermocline [6].

**Table 1:** Geographical coordinates of sampling stations with date, time, and depth of sampling at each station during the polar cruise.

St.	Latitude (N)	Longitude (W)	Date (2010/-) / Time	Seabed depth (m)	Sampling depth (m)
MS03	74°4.2′	157°18′	Jul./28 /21:58–22:23	3890	0–200, 200–500, 500–1000, 1000–1500
BN07	82°28.8′	166° 28.2′	Aug./2~3 /22:40–01:05	3610	0–200, 200–500, 500–1000, 1000–2000, 2000–3000
BN08	83°31.8′	164°03′	Aug./04 /00:00–02:30	2758	0–200, 200–500, 500–1000, 1000–2000, 2000–2400
BN11	86°4.8′	176°06′	Aug./06 /01:30–03:10	3881	0–200, 200–500, 500–1000, 1000–2000
ICE	86°55.2′	178°21.6′	(A) Aug./11/ 14:00–16:05, (B) Aug./15/ 11:40–12:35, (C) Aug./18/ 09:40–11:20	3981	0–200, 200–500, 500–1000, 1000–2000, 2000–3000

### Sampling program

Sampling was conducted from 28 July to 18 August 2010 during the late summer cruise of the Chinese R/V Xue-Long icebreaker vessel. At each station, samples were collected using a multiple opening and closing net (MultiNet, 505-μm mesh size, 0.25-m^2^ mouth opening; Hydrobios GmbH, Kiel, Germany), with a Hydrobios flowmeter mounted at the center of the net opening. The hauling velocity was approximately 0.5 m/s. Water temperature and salinity were recorded using a sensor on the net. Zooplankton samples were collected from five strata (0–200, 200–500, 500–1000, 1000–2000, and 2000–3000 m) by conducting oblique hauls at stations BN07 and ICE. Because of ice obstructions, the deepest zooplankton samples from stations MS03, BN08, and BN11 were 1500, 2400, and 2000 m, respectively. To elucidate diurnal variations, zooplankton samples were collected daily from 11 to 18 August at station ICE (Table 1). A total of 33 samples were collected from this cruise. The samples were immediately preserved in a 5% buffered formalin–sea water solution on board [32].

The investigation and zooplankton sampling are carried out in the high seas and no collection permission is required. All applicable international, national, and/or institutional guidelines for the care and use of animals were followed.

### Identification and measurement of copepods

In the laboratory, samples were split using a Folsom splitter until the subsample contained approximately 300–500 specimens. Copepod specimens were sorted and identified to the species level by using a Nikon SMZ1500 stereomicroscope. A high quality imaging system (Axio Imager M2, Zeiss, Germany) was used to observe copepod appendages. Species identification was made according to keys and references by Sars, Brodsky, Huys, and Boxshall [33–36]. The abundance of copepods was computed on the basis of the volume filtered, as estimated from the flowmeter mounted on the MultiNet equipment. All samples were deposited into the Biodiversity Collections of the Third Institute of Oceanography, State Oceanic Administration, Xiamen.

### Statistical analyses

To evaluate the distribution pattern of copepods, the data from 33 samples of 62 copepod species were computed using a cluster analysis to elucidate the relative similarities among samples. The abundance of species in each sample was used to calculate Bray–Curtis similarities before the clustering analyses. The functional test of Box and Cox [37] for data transformation was applied before the similarity analysis. The value (λ) of the power transformation for the copepod was 0.95. Therefore, log (x + 1) was applied to the logarithmic transformation of the individual densities of the copepods. Similarity analysis programs in the Paleontological Statistics (PAST) software package were used to evaluate the significance level of differences among copepod assemblages [38]. The copepod species characterizing each cluster were further identified using the Indicator Value Index (IndVal) proposed by Dufrêne and Legendre [39].

The Margalef richness and Pielou evenness indexes were used to estimate the community composition, and the Shannon–Wiener diversity index was used to evaluate the species diversity of each sample. A Pearson product moment correlation was used to estimate the correlation between copepod abundance and the temperature and salinity of water. To identify the differences in abundance among different strata and stations, a one-way analysis of variance (ANOVA) with a post hoc Tukey honestly significant difference test was applied.

## Results

### Hydrological structure of the sampling stations

**Fig 2:**
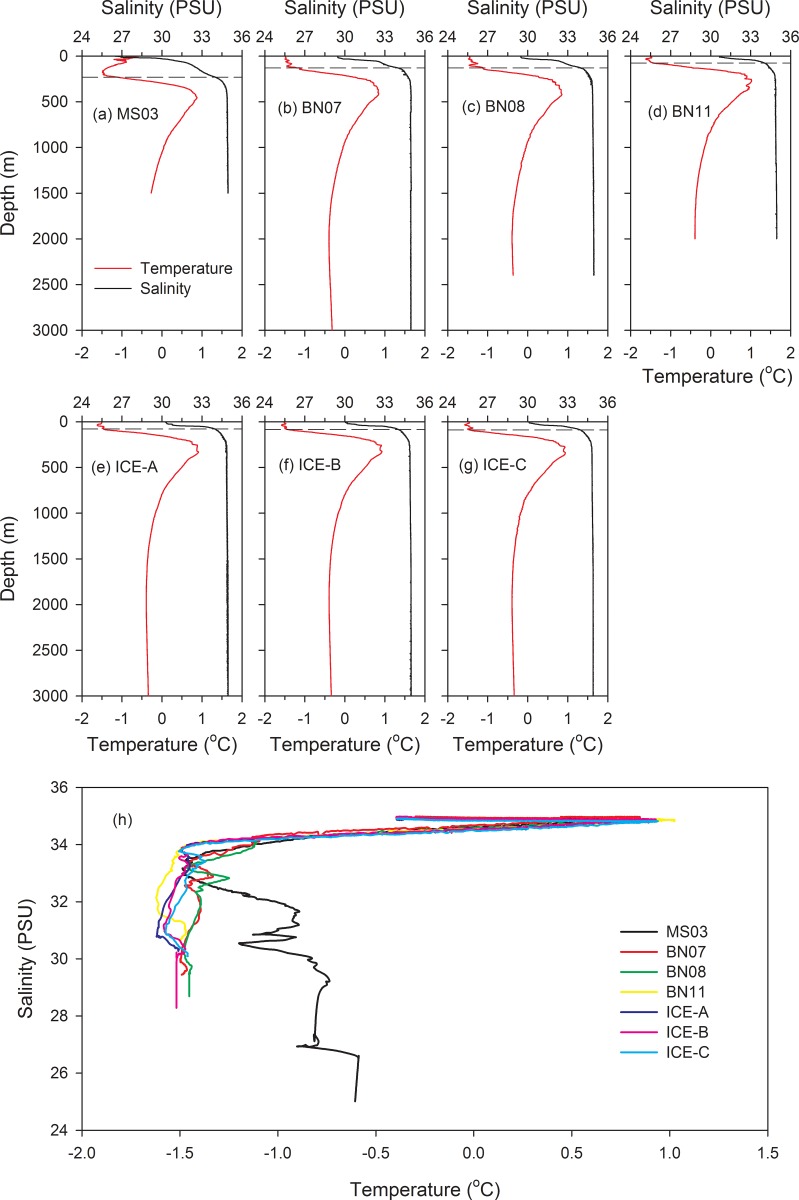
Temperature and salinity profiles above a depth of 3000 m at each sampling station. The curves were collected from stations (a) MS03, (b) BN07, (c) BN08, (d) BN11, and (e–g) ICE-A to ICE-C. Dashed lines indicate a depth of salinity of 34 PSU at each station (a–g). (h) Temperature (T) and salinity (S) of all sampling stations; the T–S diagram shows the distribution of T and S at each station.

The vertical variations of the seawater profiles provided information on the temperature and salinity for depths above 3000 m, and showed fluctuations for each sampling station (Fig 2A–2G). The depth of the thermocline was approximately 300–500 m. Temperature profiles demonstrated that the surface waters at all stations were approximately −1.5°C, except for station MS03 (−0.6°C, Fig 2A), with no clear differences between sampling stations. Sampling station MS03 was close to the Chukchi shelf, and its temperature profile showed an influence from Pacific water higher in the near-surface layer. Temperature records showed increasing values with increasing depths in the layer above 400 m. By contrast, the temperature decreased with increasing depths below approximately 400 m.

Salinities clearly exhibited an increasing trend with depth, and the highest recorded salinity occurred at approximately 200 m at most sampling stations (Fig 2). The depth recorded for a salinity value of 34 PSU showed a high variation among stations. The depths of stations MS03, BN07, BN08, BN11, and ICE were 230, 130, 130, 75, and 80–90 m, respectively. The depth of haloclines showed that the waters at stations BN07 and BN08 were characterized as mixed between station MS03 and stations BN11 and ICE. The varied pattern of salinity matched with the location of sampling stations at geospatial scales.

Vertical temperature–salinity (T–S) profiles are shown for each station in Fig 2H. Station MS03 showed a lower salinity with a high variation, which was clearly different from other stations. By contrast, the T–S curves indicate that the water masses at stations BN11 and ICE were characterized by low temperatures. The water properties of stations BN07 and BN08 showed similar patterns, with mixed characteristics between station MS03 and stations BN11 and ICE (Fig 2H).

### Copepod community structure

**Fig 3:**
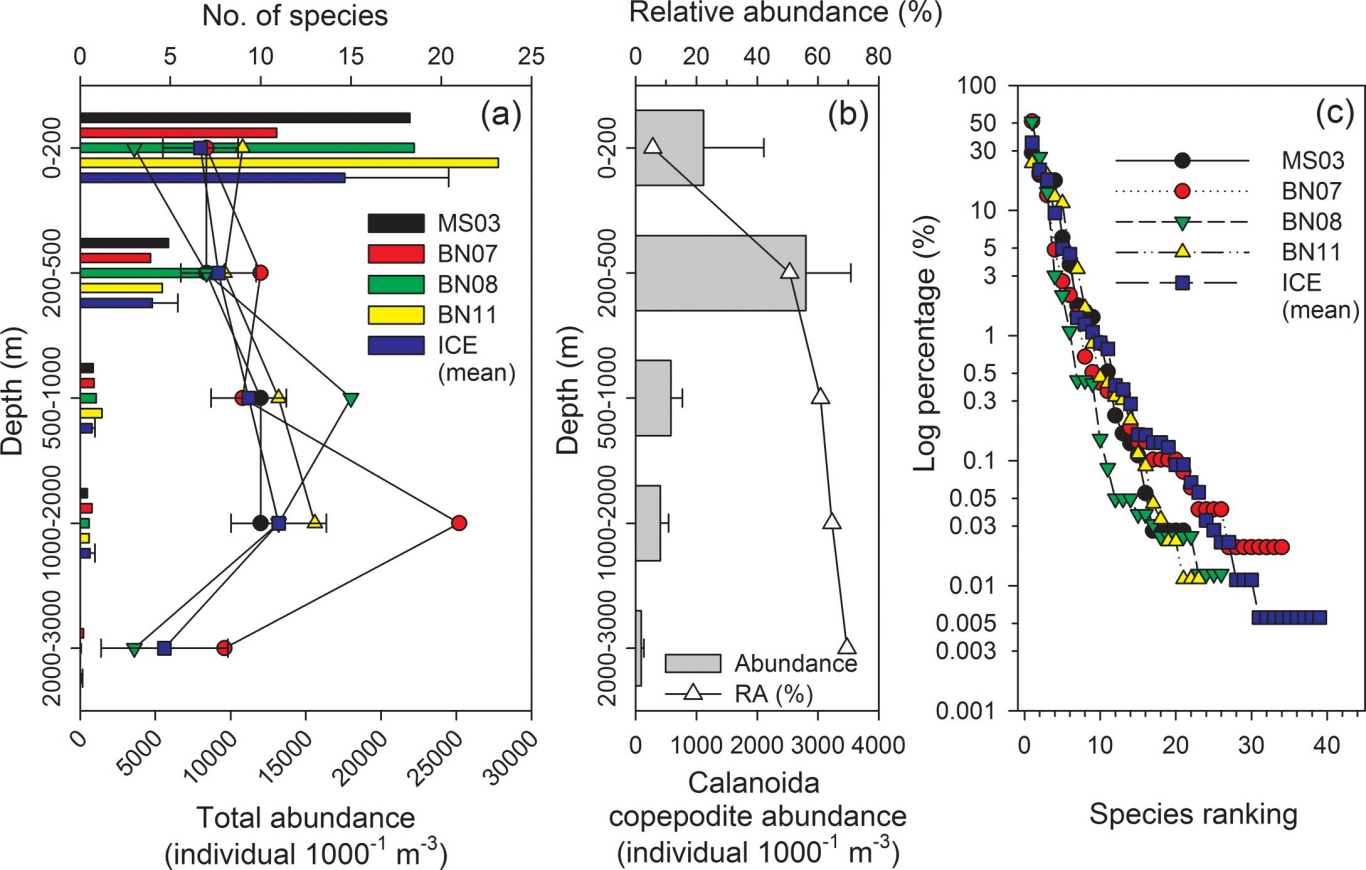
Variation of (a) copepod abundance and species number recorded from each sample, (b) average abundance and proportion (%) of Calanoida copepodite in different sampling strata, (c) rank abundance diagrams of copepods sampled from five stations. The Y-axis (abundance) is given in log 10 scales.

From a total of 33 samples from five sampling stations in the Amerasian Basin obtained in late summer 2010, a total of 55 copepod species (including 25 species that could only be identified to the generic level) and 7 taxa of copepods belonging to 28 genera, 11 families, and 2 orders were identified (Table 2). Copepod abundance and the number of species recorded over five sampling stations at each stratum are shown in Fig 3A. Integrating the data from all stations (33 samples), the maximum copepod abundance was recorded at 0–200 m at station BN11 (27,800 inds.1000^-1^ m^3^), followed by a sample at 0–200 m at station BN08 (22,200 inds.1000^-1^ m^3^), whereas the minimum abundance was recorded in a sample at 2000–3000 m at station BN08 (80 inds.1000^-1^ m^3^). The number of copepod taxa identified in each sample ranged from 3 [Station BN08 at 0–200 m and 2000–3000 m] to 21 (Station BN07 at 1000–2000 m) (Fig 3A). In particular, the abundance of Calanoida copepodites was dominant at the surface; its abundance in samples at 0–200 and 200–500 m was 1,117.1 ± 995.1 and 2,800.0 ± 739.4 (inds.1000^-1^ m^3^), respectively. We found that the proportion of Calanoida copepodites showed a contrasting pattern, increasing with sampling depth (Fig 3B). A rank abundance (%) analysis of copepod composition among the five sampling stations demonstrated geospatial variability in the structure (Fig 3C). The patterns of the rank abundance curves were relatively similar for most sampling stations, but the ICE station recorded the highest species number and identified more species with relative abundance (RA, the proportion of the number of specific species in the total number.) less than 0.1% of those at other sampling stations. The proportion of *Calanus hyperboreus* was high at stations BN07 and BN08, at over 50%.

**Table 2:** List of plankton copepods (adult and copepodite) collected in the Arctic Sea in 2010, with their mean absolute abundance, relative abundance (RA, %), and occurrence ratio (OR, %) recorded from all samples.

Station	MS03	BN07	BN08	BN11	ICE	All		
Total copepod density (Mean ± SD)	7278.7 ± 10065.5	3936.5 ± 5394.6	6442.1 ± 9423.1	8829.7 ± 12823.6	4787.2 ± 7356.5	5701.1 ± 8113.4		
Number of species identified	17	30	23	20	34	55		
Scientific classification						Mean ± SD	RA	OR
**Order Calanoida**								
Family Aetideidae								
*Aetideopsis armata* (Boeck, 1872)	0	0	1.60	0	0	0.24 ± 1.39	<0.01	3.03
*Aetideopsis multiserrata* (Wolfenden, 1904)	0	4.00	0.80	2.00	0	0.97 ± 3.75	0.02	9.09
*Aetideopsis rostrata* Sars, 1903	8.00	1.60	28.27	28.67	13.60	15.15 ± 42.19	0.27	30.30
*Batheuchaeta lamellata* Brodsky, 1950	0	0	0	0	0.27	0.12 ± 0.7	<0.01	3.03
*Bradyidius* sp.	0	0	0	0	0.53	0.24 ± 1.39	<0.01	3.03
*Chiridiella abyssalis* Brodsky, 1950	0	3.20	2.40	1.00	1.07	1.45 ± 2.8	0.03	24.24
*Chiridiella reductella* Markhaseva, 1996	0	0.80	0	0	0	0.12 ± 0.7	<0.01	3.03
*Chiridius obtusifrons* Sars, 1902	0	0.80	0	0	0	0.12 ± 0.7	<0.01	3.03
*Chiridius* sp.	0	0	0	0	0.27	0.12 ± 0.7	<0.01	3.03
*Euchirella* sp.	2.00	0	0	0	0	0.24 ± 1.39	<0.01	3.03
*Gaetanus brevispinus* (Sars, 1900)	0	1.60	0	0	0.27	0.36 ± 1.54	0.01	6.06
*Gaetanus* sp.1	0	57.33	0	0	7.64	12.16 ± 47.38	0.21	21.21
*Gaetanus* sp.2	0	0	9.60	0	7.73	4.97 ± 19.05	0.09	12.12
*Gaetanus* sp.3	0	0.80	0	0	0.27	0.24 ± 0.97	<0.01	6.06
*Gaetanus tenuispinus* (Sars, 1900)	0	2.40	69.87	27.00	17.78	22.3 ± 53.95	0.39	30.30
Family Augaptilidae								
*Augaptilus glacialis* Sars G.O., 1900	2.00	0	0	3.00	0	0.61 ± 2.47	0.01	6.06
*Augaptilus* sp.	0	0	0	0	6.13	2.79 ± 6.96	0.05	15.15
*Centraugaptilus* sp.	0	26.67	0	0	0	4.04 ± 23.21	0.07	3.03
*Euaugaptilus hyperboreus* Brodsky, 1950	2.00	0	3.20	0	4.44	2.75 ± 11.88	0.05	9.09
*Euaugaptilus* sp.	0	0	0.80	0	0	0.12 ± 0.7	<0.01	3.03
*Haloptilus acutifrons* (Giesbrecht, 1892)	0	16.53	1.60	1.00	1.60	3.6 ± 12.04	0.06	21.21
*Haloptilus longicirrus* Brodsky, 1950	0	0	0	0	0.53	0.24 ± 1.39	<0.01	3.03
Family Bathypontiidae								
*Temorites brevis* Sars, 1900	37.33	7.20	1.60	76.33	37.51	32.16 ± 78.09	0.56	48.48
Family Calanidae								
*Calanus finmarchicus* (Gunnerus, 1770)	0	0	0	36.67	0.53	4.69 ± 17.84	0.08	9.09
*Calanus glacialis* Jaschnov, 1955	1302.00	520.80	5.60	1806.00	213.60	553.58 ± 1581.88	9.71	45.45
*Calanus hyperboreus* Kröyer, 1838	2101.33	2024.00	3292.67	2109.33	1011.64	1775.78 ± 3691.78	31.15	90.91
Family Eucalanidae								
*Eucalanus bungii* Giesbrecht, 1892	0	0	0	18.67	0	2.26 ± 11.65	0.04	6.06
*Paraeuchaeta glacialis* (Hansen, 1887)	63.33	0.80	0.80	10.00	51.11	32.36 ± 89.04	0.57	24.24
*Paraeuchaeta* sp.	0	13.33	0	0	0.27	2.14 ± 11.6	0.04	6.06
Family Heterorhabdidae								
*Heterorhabdus norvegicus* (Boeck, 1872)	126.67	0.80	1.60	0	1.33	16.32 ± 76.95	0.29	21.21
*Heterorhabdus* sp.1	0	0	0	0	<0.01	< 0.01	<0.01	3.03
*Heterorhabdus* sp.2	0	0	0	0	6.67	3.03 ± 17.41	0.05	3.03
*Paraheterorhabdus compactus* (Sars, 1900)	0	1.60	0	2.00	0.27	0.61 ± 2.03	0.01	9.09
Family Lucicutiidae								
*Lucicutia anomala* Brodsky, 1950	0	0	0	0	0.27	0.12 ± 0.7	<0.01	3.03
*Lucicutia polaris* Brodsky, 1950	4.00	4.00	2.40	8.00	3.20	3.88 ± 7.65	0.07	27.27
*Lucicutia* sp.1	0	0.80	0	0	0	0.12 ± 0.7	<0.01	3.03
*Lucicutia* sp.2	0	0	2.00	0	2.67	1.52 ± 7.12	0.03	6.06
Family Metridinidae								
*Metridia longa* (Lubbock, 1854)	440.67	190.67	900.93	1009.67	457.16	548.99 ± 1045.29	9.63	81.82
*Metridia princeps* Giesbrecht, 1889	0	0.80	0	0	0	0.12 ± 0.7	<0.01	3.03
Family Phaennidae								
*Onchocalanus* sp.	0	0	0	0	0.27	0.12 ± 0.7	<0.01	3.03
Family Scolecitrichidae								
*Amallothrix* sp.	0	14.13	0	0	0	2.14 ± 11.6	0.04	6.06
*Lophothrix* sp.	0	0	0.80	0	0	0.12 ± 0.7	<0.01	3.03
*Mixtocalanus* sp.	0	13.33	26.67	4.00	19.11	15.23 ± 36.18	0.27	21.21
*Scolecithricella* minor (Brady, 1883)	114.00	4.00	0	0	0	14.42 ± 76.5	0.25	12.12
*Scolecithricella* sp.	0	0	0	1.00	0	0.12 ± 0.7	<0.01	3.03
*Scaphocalanus* copepodite	0	0	0	0	<0.01	<0.01	<0.01	3.03
*Scaphocalanus magnus* (T. Scott, 1894)	16.67	106.67	134.93	146.33	66.40	86.55 ± 149.35	1.52	60.61
*Scaphocalanus polaris* Brodsky, 1950	0	0	0	0	1.07	0.48 ± 1.94	0.01	6.06
*Scaphocalanus* sp.1	2.00	0	0	0	0	0.24 ± 1.39	<0.01	3.03
*Scaphocalanus* sp.2	2.00	20	0	41.00	59.11	35.11 ± 73.33	0.62	45.45
*Scaphocalanus* sp.3	12.00	0.80	3.20	0	0	2.06 ± 6.64	0.04	12.12
*Scaphocalanus* sp.4	0	0	3.20	0	0	0.48 ± 2.79	0.01	3.03
Family Spinocalanidae								
*Spinocalanus horridus* Wolfenden, 1911	0	1.60	0	0	0	0.24 ± 1.39	<0.01	3.03
*Spinocalanus magnus* Wolfenden, 1904	268.00	82.67	193.87	395.00	237.42	230.18 ± 437.77	4.04	72.73
**Order Mormonilloida**								
Family Mormonillidae								
*Mormonilla* sp.	0	0	0	0	6.67	3.03 ± 17.41	0.05	3.03
**Copepodite**								
*Aetideopsis* copepodite	10.00	0	0	0	0	1.21 ± 6.96	0.02	3.03
*Euaugaptilus* copepodite	0	0	1.60	0	0	0.24 ± 1.39	<0.01	3.03
*Calanus* copepodite	1400	5.60	0	1667.67	1663.38	1128.77 ± 2771.86	19.80	48.48
*Paraeuchaeta* copepodite	102.00	4.00	28.27	300	41.87	72.65 ± 237.46	1.27	33.33
*Heterorhabdus* copepodite	0	0	0	0	4.44	2.02 ± 11.61	0.04	3.03
*Spinocalanus* copepodite	0	5.60	0	0	0.27	0.97 ± 4.9	0.02	6.06
Calanoida copepodite	1262.67	797.60	1723.87	1135.33	838.84	1054 ± 1118.8	18.49	96.97

Among all samples, the five most abundant taxa were *Calanus hyperboreus* (RA: 31.15%), *Calanus* copepodite (RA: 19.80%), Calanoida copepodite (RA: 18.49%), *Calanus glacialis* (RA: 9.71%), and *Metridia longa* (RA: 9.63%). In terms of frequency of occurrence, the following four species occurred in > 60% samples: *Calanus hyperboreus* (90.91%), *Metridia longa* (81.82%), *Spinocalanus magnus* (72.73%), and *Scaphocalanus magnus* (60.61%) (Table 2). A total of 9 species were identified from five sampling stations: *Aetideopsis rostrata*, *Temorites brevis*, *Calanus glacialis*, *Calanus hyperboreus*, *Paraeuchaeta glacialis*, *Lucicutia polaris*, *Metridia longa*, *Scaphocalanus magnus*, and *Spinocalanus magnus*. Twenty-six species of copepod (including 14 species that were solely identified to the generic level and 4 taxa of copepodites) were only found in a single sample, although their occurrence rate among all samples was 3.03% (Table 2).

**Fig 4:**
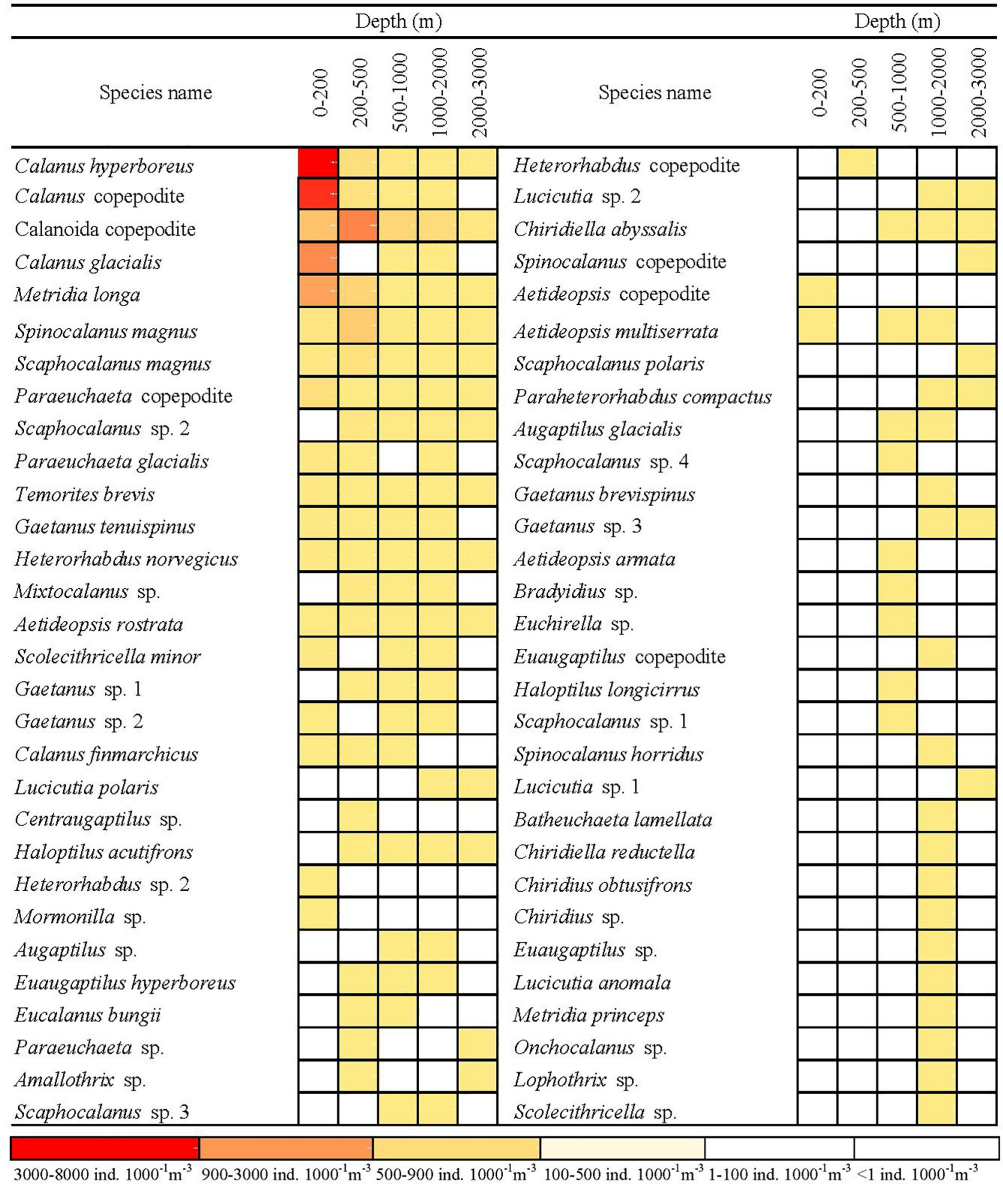
Abundance and distribution of each copepod species from five sampling strata.

The abundance rank combined with an occurrence rate analysis of each copepod species showed different distribution patterns by using a vertical scale (Fig 4). Most species of copepod displayed specific habitat depths. Ten species were identified from five sampling strata: *Calanus hyperboreus*, *Metridia longa*, *Spinocalanus magnus*, *Scaphocalanus magnus*, *Paraeuchaeta* copepodite, *Temorites brevis*, *Heterorhabdus norvegicus*, and *Aetideopsis rostrata*. Some species demonstrated a clear distribution pattern in Arctic surface waters, such as the *Calanus hyperboreus*, *C*. *glacialis*, *Eucalanus bungii*, *Heterorhabdus norvegicus*, *Metridia longa*, *Paraeuchaeta glacialis*, *Scaphocalanus magnus*, *Scolecithricella minor*, and *Spinocalanus magnus*; each had a high abundance and occurrence rate in samples at the surface. Among all samples, many species were absent in upper water layers: *Aetideopsis armata*, *Augaptilus glacialis*, *Augaptilus* sp., *Bradyidius* sp., *Euchirella* sp., and *Haloptilus longicirrus* were recorded only in samples below 500 m. In addition, there were 6, 13, and 3 taxa that preferred deeper strata and were identified only in samples at 500–1000, 1000–2000, and 2000–3000 m, respectively.

**Fig 5:**
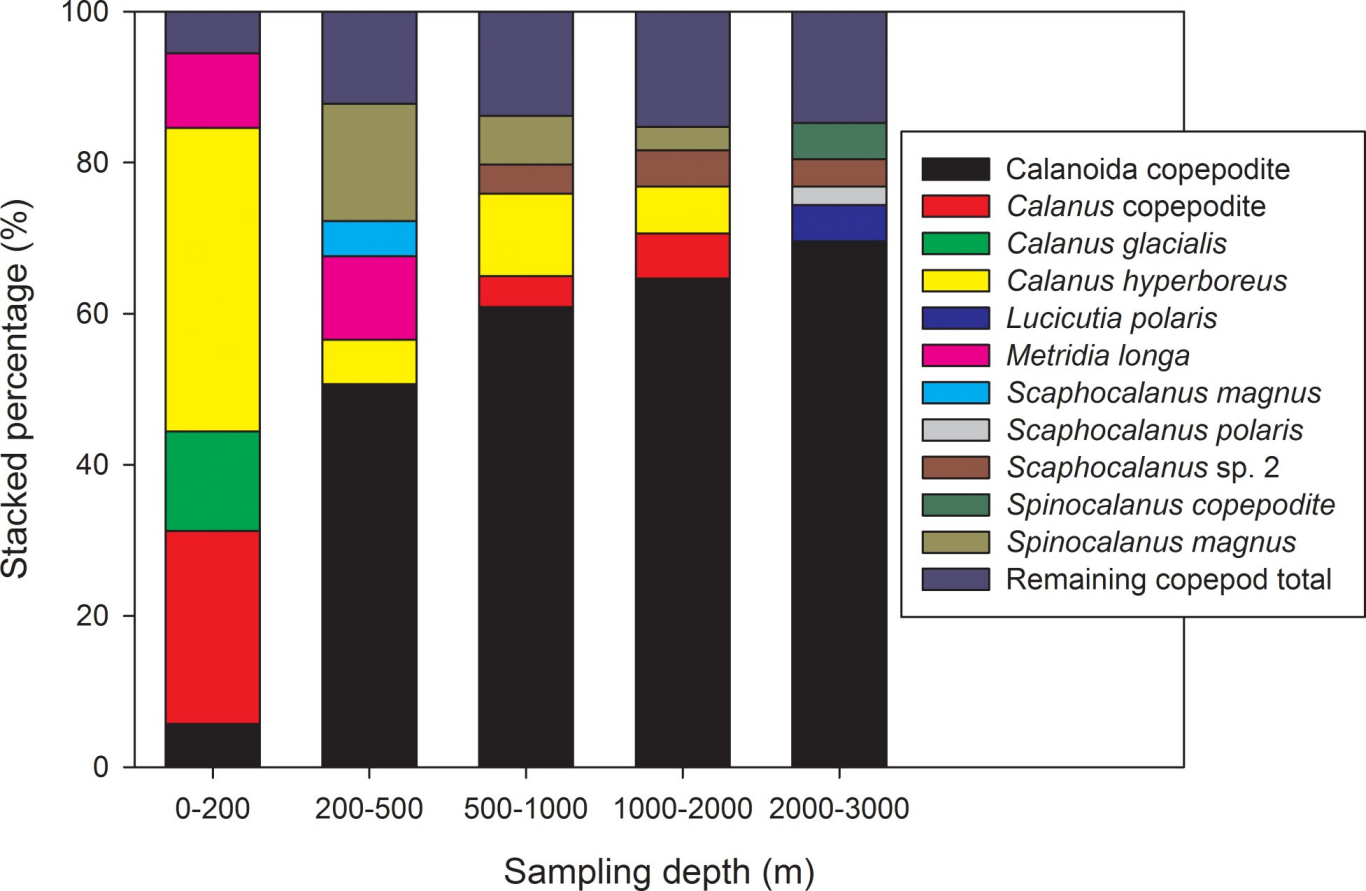
Relative abundance of the five most abundant copepod species identified from five different sampling stations.

The rank of RAs of the top five dominant species at each sampling station showed geospatial variation (Fig 5). The dominant taxa at each station varied: Calanoida copepodites were dominant in samples deeper than 200 m, with an RA higher than 50%; it was particularly dominant in samples at 2000–3000 m, with an RA of 69.58%. *Calanus* copepodite was dominant at 0–200 m (RA: 25.54%) and in samples at 500–1000 and 1000–2000 m, with an RA of 4.08% and 6.0%, respectively. *Calanus hyperboreus* ranked second in samples at 0–200 (RA: 40.16%) and 500–1000 m (RA: 10.92%); its RA was higher in samples collected from depths above the 2000-m stratum (RA > 5.8%). *Metridia longa* was dominant in the surface strata of 0–200 and 200–500 m. *Spinocalanus magnus* exhibited relatively high RA values at 200–500 (15.52%), 200–1000 (6.48%), and 1000–2000 m (3.09%). *Calanus glacialis* (RA: 13.18%), *Scaphocalanus magnus* (RA: 4.66%), and *Lucicutia polaris* (RA: 2.41%) were dominant only in the 0–200, 200–500, and 2000–3000-m strata, respectively (Fig 5).

### Hierarchical classification

**Fig 6:**
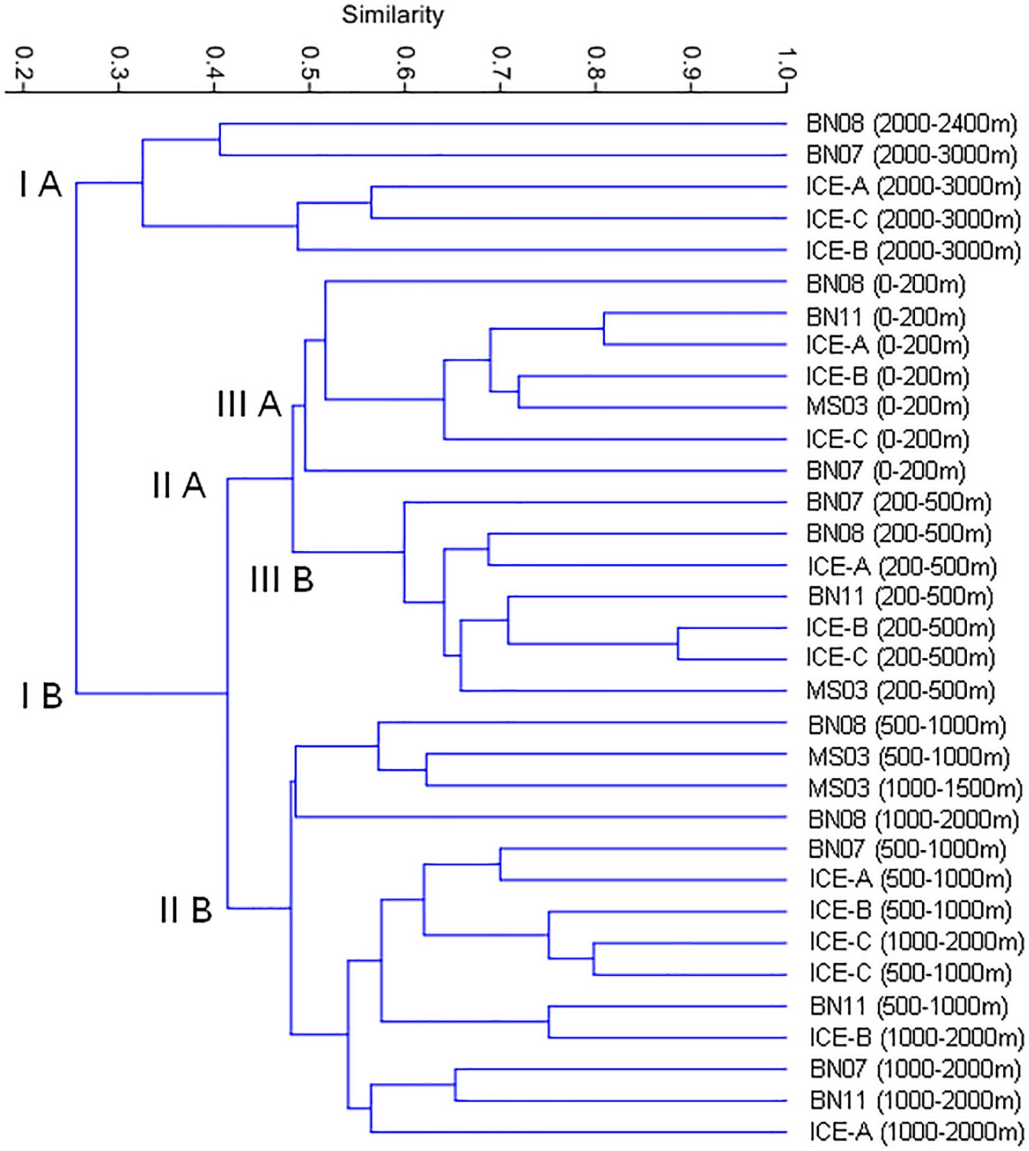
Clustering dendrogram of different samples by using Bray–Curtis similarities of copepod composition among 33 samples.

A copepod assemblage analysis based on Bray–Curtis similarities showed that the stratum variations in community structure were separated (Fig 6). Table 3 provides the copepod composition and distribution for the 33 samples at five sampling depths. At the highest grouping level, five samples with a lower abundance of copepods collected at 2000–3000 m were separated into Group I A (Fig 6). The three major copepod species of Group I A were Calanoida copepodites (IndVal: 69.58%), belonging to *Lucicutia polaris* (IndVal: 2.89%), and *Spinocalanus* spp. copepodites (IndVal: 1.93%) (Table 3). The second hierarchical level separated the samples collected at 0–200 and 200–500 m (Group II A), and at 500–1000 and 1000–2000 m (Group II B). The samples collected in Group II B were characterized by the dominance of Calanoida copepodites (IndVal: 62.36%), *Calanus hyperboreus* (IndVal: 9.04%), and *Spinocalanus magnus* (IndVal: 5.13%). The third hierarchical level was restricted to a differential community pattern of copepod species composition, separating samples by the depths of 0–200 (Group III A) and 200–500 m (Group III B). In Group III A, *Calanus hyperboreus* (IndVal: 40.16%) was followed by *Calanus* copepodite (IndVal: 18.24%) and *Calanus glacialis* (IndVal: 11.30%). Group III B showed three dominant species: Calanoida copepodite (IndVal: 50.69%), *Spinocalanus magnus* (IndVal: 15.52%), and *Metridia longa* (IndVal: 11.03%) (Table 3).

**Table 3:** Indicator species and index values (%) of each copepod species with a value exceeding 1% for each cluster identified using Bray–Curtis cluster analysis (Fig 6).

	Cluster group			
Indicator species	IA	II B	III A	III B
Calanoida copepodite	69.58	62.36	4.87	50.69
*Calanus glacialis* Jaschnov, 1955			11.30	
*Calanus hyperboreus* Kröyer, 1838		9.04	40.16	5.86
*Calanus* copepodite		2.77	18.24	1.70
*Lucicutia polaris* Brodsky, 1950	2.89			
*Metridia longa* (Lubbock, 1854)		1.25	9.92	11.03
*Scaphocalanus magnus* (T. Scott, 1894)				4.66
*Scaphocalanus* sp.2	1.45	3.02		
*Spinocalanus* copepodite	1.93			
*Spinocalanus magnus* Wolfenden, 1904		5.13		15.52
*Temorites brevis* Sars, 1900		1.06		

### Statistical analysis

**Fig 7:**
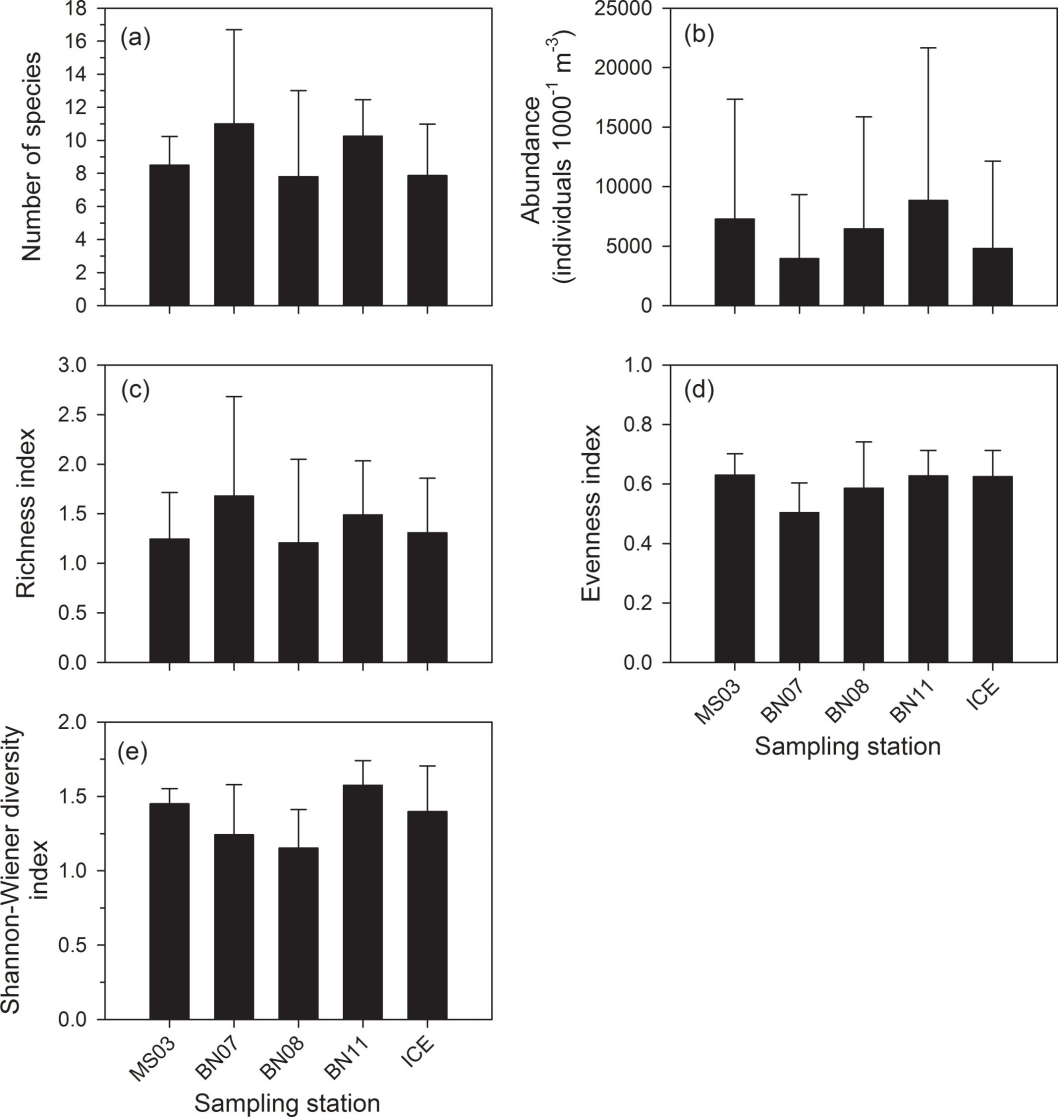
Comparisons of (a) abundance, (b) number of species, (c) indices of richness, (d) evenness, and (e) diversity from 5 sampling stations by using one-way ANOVA followed by the Tukey test.

Multiple comparisons of mean values among the five sampling stations were conducted using a one-way ANOVA followed by the Tukey test (Fig 7). The results revealed that the effects of geospatial variability on the number of species (Fig 7A), abundance (Fig 7B), and the indexes of richness (Fig 7C), evenness (Fig 7D), and Shannon–Wiener diversity (Fig 7E) were not significant (*p* > 0.05).

**Fig 8:**
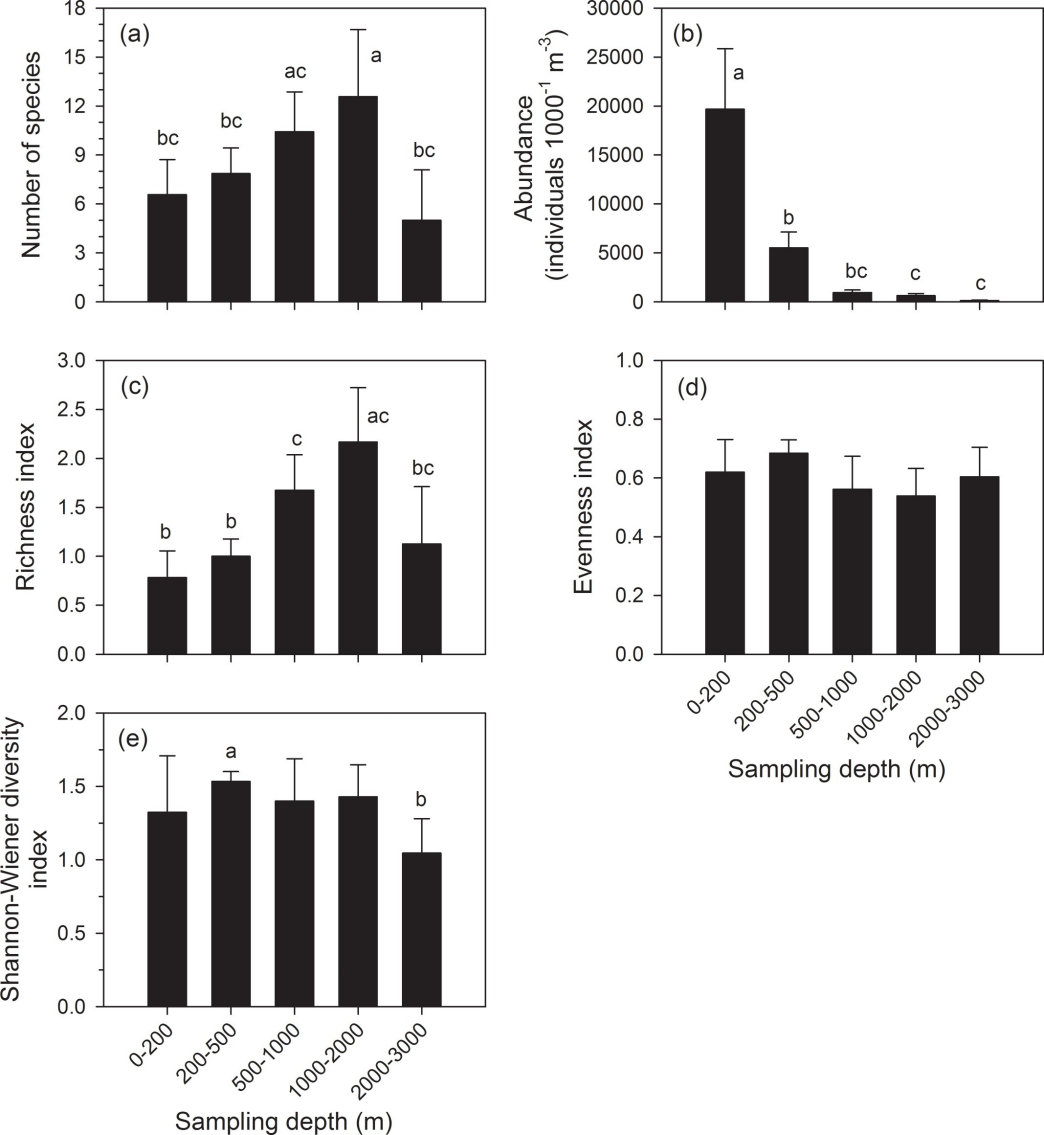
Comparisons of (a) abundance, (b) number of species, (c) indices of richness, (d) evenness, and (e) diversity from five sampling strata by using one-way ANOVA followed by the Tukey test. The superscripts (a, b, c) denote significant differences (*p* < 0.05, one-way ANOVA) among five sampling strata.

Across the five sampling strata, the number of species found at 1000–2000 m was significantly higher than the numbers found at 0–200 (*p* = 0.003), 200–500 (*p* = 0.028), and 2000–3000 m (*p* = 0.001); the number of species at 500–1000 m was significantly higher than at 2000–3000 m (*p* = 0.019) (Fig 8A). The total abundance of copepod in samples at 0–200 m was significantly higher than in other strata (*p* < 0.001); in samples at 200–500 m, the total abundance was significantly higher than at 1000–2000 m (*p* = 0.034) and 2000–3000 m (*p* = 0.032) (Fig 8B). The index of richness at 1000–2000 m was significantly higher than at 0–200 (*p* < 0.001), 200–500 (*p* < 0.001), and 2000–3000 m (*p* = 0.001); at 500–1000 m, the index of richness was significantly higher than at 0–200 (*p* = 0.003) and 200–500 m (*p* = 0.003) (Fig 8C). The index of evenness was not significantly different among the five sampling strata (*p* > 0.05) (Fig 8D). The Shannon–Wiener diversity index was significantly higher at 200–500 m than at 2000–3000 m (*p* = 0.027) (Fig 8E).

## Discussion

### Copepod assemblage structure

Copepods play a crucial role in pelagic food webs because of their abundance and as consumers of the primary production [40, 41]. Several studies on zooplankton in the Arctic have reported that copepods were the most substantial taxa in terms of species number, abundance, and biomass [25, 42]. Their results demonstrated that copepod assemblages were strongly affected by Pacific, Atlantic, and fresh water masses among sampling stations at different depths. Similarly, the composition of the copepod community was affected by different water masses in the Arctic region [14]. Furthermore, the copepod community structure was highly correlated with water mass properties, particularly the temperature in the bottom zone [43].

This study found two species recorded for the first time in this region: *Haloptilus longicirrus* and *Batheuchaeta lamellata*. *Haloptilus longicirrus* was reported in the Shipunsky Cape by Brodsky [34]. After three decades, Buchanan and Sekerak [44] recorded one immature individual (copepodite V) of *H*. *longicirrus* from a sample below the depth of 250 m in Baffin Bay. *Haloptilus longicirrus* was originally described from the northwest Pacific. It is widely distributed in the northeastern Atlantic, the Antarctic and western parts of the Indian Ocean [45, 46], the north Atlantic, the Caribbean, the Gulf of Mexico [47], and the Kuroshio Current of the western Pacific [48]. In this study, *H*. *longicirrus* was only found at station ICE at a depth of 500–1000 m. Our results confirmed that the habitat of *H*. *longicirrus* was at a depth of approximately 600 m to below 1000 m [47]; however, Hsiao et al. [48] found it to be in a shallower zone above the 200-m depth. This study recorded *B*. *lamellate* at the ICE station at a depth of 1000–2000 m. The location of this station is far from the location reported by Brodsky [34] of *B*. *lamellata* in the northwestern Pacific. Previous reports documented that both species are recorded in deep water [34, 44–48], indicating that they inhabited the cold water environment.

Previous studies revealed that *Calanus glacialis*, *Calanus hyperboreus*, and *Metridia longa* were the dominant species in the Arctic Ocean [24, 25, 42]. This study found most of these species at the sampling stations at different developmental stages, from early copepodites to adults. This confirmed a similar phenomenon in the Canada Basin [49]. The high proportion of copepodites in most samples indicated that these species had successfully adapted and bred in the Arctic Ocean. In addition, both *C*. *hyperboreus* and *C*. *glacialis* were found to reproduce in the Arctic Ocean [42]. The dominant species of this study were *C*. *hyperboreus*, *M*. *longa*, *C*. *glacialis*, *Spinocalanus magnus*, and *Scaphocalanus magnus*, excluding the taxon copepodite. The life span of *C*. *hyperboreus* is estimated to range from 2 to 6 years, and it is an indicator species in Arctic waters [50]. This species plays a herbivorous, low-level consumer role in the Arctic marine ecosystem [20]. In the present study, *C*. *hyperboreus* was found in samples collected between 2000 m and the surface. Previous studies reported a seasonal migration of *C*. *hyperboreus*, distributed at depths below 1000 m from September to March and migrating to the surface layer in May and June [17, 50]. Our results confirmed the seasonal vertical migration of this species in the thermally stratified Amundsen Gulf [51].

The Arctic shelf species *C*. *glacialis* and *M*. *longa* are both bioindicator species in Arctic waters [17, 52]. In this research, most *C*. *gracilis* were recorded at the surface layer, but a few individuals were found in samples collected at a depth of 500 to 2000 m. *M*. *longa* usually resides at an intermediate depth without a clear seasonal vertical migration in the Arctic [51]. This study identified *M*. *longa* in samples collected from the surface to 3000 m; the highest density was in the water layer above 500 m. This species is an essential omnivorous and detritivorous consumer that feeds on diverse food particles in the Arctic ecosystem. It might feed on the buoyant eggs of *C*. *hyperboreus* in winter [53].

The boreal copepod *Calanus finmarchicus* is generally distributed in Atlantic waters, and is a common and abundant species in the eastern Arctic where it is affected by water masses from the Atlantic Ocean [54–57]. The present study only recorded *C*. *finmarchius* at stations BN11 and ICE in the 100 to 1000 m water layer of the Makarov Basin. Conover and Huntley [58] revealed a large number of *C*. *finmarchicus* through the Fram Strait and the Barents Sea shelf injected into the Arctic, with the density decreasing from west to east along the inflow of Atlantic waters [55, 56, 58]. By contrast, *C*. *finmarchicus* was reportedly rare in the Makarov Basin. Only a few specimens were found in the western Makarov Basin, which is affected by the countercurrent of Atlantic inflows [23, 59, 60]. Our results confirmed that *C*. *finmarchicus* is completely absent in the Canada Basin [15].

Copepod communities in the Arctic Ocean are generally affected by several factors such as the seasonality of light regimes, ice cover ratios, the advection of waters from adjacent seas [61], and seasonal vertical migrations [51]. To survive against the extreme light and ice cover, copepods have evolved different life strategies to maintain their populations in the Arctic [51, 62]. The present study found abundant copepodites distributed in the water layer above 500 m and a high proportion in deeper layers (Fig 3B). This is similar to a finding that copepodites of the dominant species *Calanus hyperboreus*, *C*. *glacialis*, *C*. *hyperboreus*, *C*. *finmarchicus*, and *Metridia longa* had a high proportion in open-water (71.15%) and ice-covered (76.86%) regions [61]. Five copepod species (*Calanus glacialis*, *C*. *hyperboreus*, *Metridia longa*, *Microcalanus pygmaeus*, and *Oithona similis*) in the western Arctic Ocean were shown to have two general life history strategies: (1) sustained reproduction with all life stages present throughout the year and a constant depth distribution, and (2) pulsed reproduction with overlapping cohorts and an ontogenetic redistribution of preferred depths throughout the year [42]. Consequently, a high proportion of copepodites among samples was found in the present study.

### Geospatial variation and vertical distribution

Geospatial variability in the community structure of zooplankton has been examined in different regions, including a river system [63], the southeastern Bering Sea [64], the East China Sea [65], and the South China Sea [66, 67]. To reveal the geospatial variation in the Arctic Ocean, we compared the copepod composition between stations MS03 and ICE, which have the longest distance between them of approximately 1400 km. The composition and dominant copepods of these two stations revealed comparative differences in species richness and proportions (Tables 2 and 4). As for the taxonomic results, we found that *Augaptilus glacialis*, *Gaetanus brevispinus*, *Heterorhabdus norvegicus*, and *Scolecithricella minor* were only recorded in the samples at station MS03. By contrast, *Batheuchaeta lamellate*, *Calanus finmarchicus*, *Chiridiella abyssalis*, *Gaetanus tenuispinus*, *Haloptilus acutifrons*, *Haloptilus longicirrus*, *Lucicutia anomala*, *Paraheterorhabdus compactus*, and *Scaphocalanus polaris* were only found in samples from station ICE (Table 4). The samples collected from stations MS03 and ICE show distinguishingly different copepod abundances and species richness. The locations of stations MS03 and ICE were affected by North Pacific [68, 29] and Atlantic (6, 30) waters, respectively. Ice cover might be unfavorable for copepod development. In our study, the RA of copepodite was higher at ICE station than at MS03 station (Table 4). To date, *S*. *polaris* has only been recorded in the Laptev Sea [61]. Several reports have suggested that copepods could be used as bioindicators to track water mass transport [67, 69]. Such copepods might have the potential to be used bioindicators for water mass movement in the Arctic Ocean. Furthermore, the composition and structure of copepods strongly demonstrated geospatial variation in the Arctic Ocean. The present study confirmed that copepod abundance and species composition varied significantly at horizontal and vertical scales [64–67].

**Table 4:** Dominant species and specific copepod species found at stations MS03 and ICE. RA is relative abundance (%), DS is depth of samples.

Sampling station	Dominant species (RA)	Only found species (DS)
MS03	*Calanus hyperboreus* (28.87), *Calanus* copepodite (19.23), *Calanus glacialis* (17.89), Calanoida copepodite (17.35), *Metridia longa* (6.05), Remaining species total (10.61)	*Augaptilus glacialis* (500-100m), *Gaetanus brevispinus* (1000-2000m), *Heterorhabdus norvegicus* (0-200m, 200-500m), *Scolecithricella minor* (0-200m, 500-1000m, 1000-2000m)
ICE	*Calanus* copepodite (34.75), *Calanus hyperboreus* (21.13), Calanoida copepodite (17.52), *Metridia longa* (9.55), *Spinocalanus magnus* (4.96), Remaining species total (12.09)	*Batheuchaeta lamellate* (1000-2000m), *Calanus finmarchicus* (500-100m), *Chiridiella abyssalis* (500-1000m, 1000-2000m), *Gaetanus tenuispinus* (0-200m, 200-500m), *Haloptilus acutifrons* (1000-2000m, 2000-3000m), *Haloptilus longicirrus* (500-1000m, 1000-2000m, 2000-3000m), *Lucicutia anomala* (1000-2000m), *Paraheterorhabdus compactus* (2000-3000m), *Scaphocalanus polaris* (2000-3000m)

The present study recorded a high density of copepods aggregated in samples at 0–200 m at all stations. This pattern has been reported in several studies [15, 42, 70]; their results suggested that the distribution of zooplankton has an annual cycle in the Arctic. A high density of zooplankton in the upper layer is typically observed during the summer period. This may be correlated with the food supply from ice algae. Saiz [19] pointed out that the maximum copepod density and biomass was often close to the fluorescence maximum in the Arctic Ocean. By contrast, the distribution pattern of copepods was not evident at the horizontal scale across sampling stations. Dunbar and Harding [49] suggested a distribution pattern of copepods without clear boundaries among three main water masses in the Arctic Ocean. Our results confirmed their report; some copepod species were identified at all stations, indicating lower boundary effects of spatial distribution patterns in the Arctic Ocean.

In our research, the number of copepod species and richness index were higher in the 1000–2000 m samples than at other layers. Previous reports revealed that copepod diversity increased with depth in the Arctic Ocean [14, 23, 54, 71–73]. Kosobokova [15] found that copepod assemblages in the Arctic were dominated by a few species in surface water and the diversity, evenness and richness indices were lower in surface waters. However, these reports demonstrated that most diversity and evenness occurred at mid-depths (~200 to 500 meters), and richness of species peaked slightly deeper at about 500-1000m. The latter distribution patter was the same as in our present study.

## Conclusions

Global warming is rapidly and severely affecting the Arctic. Climate change has caused rising temperatures, melting ice sheets, and a loss of sea ice. Therefore, changes in the Arctic have an impact on local people and ecosystems [74]. Zooplankton plays a critical role in ocean ecosystems, and diverse communities are sensitive to their environment and to climate change [75]. Thus, monitoring the changes in zooplankton and marine biodiversity is important for the understanding of thermal adaptation to climate changes in the Arctic [76]. The present study suggests that long-term tracking of the dynamics in zooplankton assemblage structures is crucial to evaluate the potential effects of global warming on marine ecosystems in the Arctic. In conclusion, this study presents four key results: (1) The abundance of copepods at 0–200 m was significantly higher than in other strata, and the number of species was high in the 1000–2000 m stratum. (2) Water strata provided diverse and stable environments, leading to significant differences in the vertical and spatial composition distribution of planktonic copepods during the study period in the Arctic. (3) The first biogeographical distribution record of *Haloptilus longicirrus* and *Batheuchaeta lamellate* confirms the water movement within the North Pacific, Arctic Ocean, and Northern Atlantic. (4) The species composition of copepods exhibits geospatial differences because of the influence of different water masses: the North Pacific and Atlantic waters.

## Supporting information

S1 File Supporting information file provides all taxonomic result data. (XLSX)
